# Infrared thermography in the diagnosis of musculoskeletal injuries: A protocol for a systematic review and meta-analysis

**DOI:** 10.1097/MD.0000000000023529

**Published:** 2020-12-04

**Authors:** Xiaoyu Li, Yajun Zhang, Haiju Sun, Yongliang Jiang, Jiali Lou, Xiaofen He, Jianqiao Fang

**Affiliations:** a The Third Affiliated Hospital of Zhejiang Chinese Medical University, Hangzhou City, Zhejiang Province; b Department of Neurobiology and Acupuncture Research, The Third Clinical Medical College, Zhejiang Chinese Medical University, Key Laboratory of Acupuncture and Neurology of Zhejiang Province, Hangzhou, China.

**Keywords:** diagnostic accuracy, infrared thermography, meta-analysis, musculoskeletal injuries, protocol

## Abstract

**Background:**

Musculoskeletal injuries (MSDs) have become a major public health problem worldwide. Current diagnosis techniques for MSDs are often associated with radiation exposure, expensive cost, or contraindication. Infrared thermography (IRT) is becoming a proposed tool to assist in diagnosing MSDs, but current evidence is inconclusive. Thus, herein we aimed to evaluate the diagnostic accuracy of IRT for MSDs.

**Methods:**

We will search EMBASE, MEDLINE, EBSCO, Cochrane Library, SCOPUS, Web of Science, CNKI, SinoMed, and Wangfang. Two researchers will independently screen eligible studies. Study quality will be evaluated based on the Quality Assessment of Diagnostic Accuracy Studies (QUADAS-2) tool. Data synthesis will be completed using STATA 14.0 software. A bivariate random-effects analysis will be utilized to estimate the pooled estimation of the diagnostic odds ratio (DOR) and the summary receiver operating characteristics (SROC) curve. Subgroup analyses will be performed to determine heterogeneity sources.

**Results:**

This systematic review and meta-analysis will provide reliable evidence about the diagnostic accuracy of IRT for MSDs.

**Conclusion:**

The conclusion of this study will be published in a peer-reviewed journal.

**Ethics and Communication:**

Given that this is a systematic review of published research, patient consent and ethical approval are not relevant. The findings of this study will be disseminated through conference presentations and publication in peer-reviewed journals.

**Prospero registration number:**

CRD42020184867.

## 1 Introduction

Musculoskeletal injuries (MSDs), such as fractures, dislocations, sprains, contusions, and compartment syndrome, are among the most disabling diseases affecting Americans.^[1]^ According to an epidemiological survey, the proportion of MSDs increased from 28.0% in 1996 to 1998 to 33.2% in 2009 to 2011, the number increased from 76.0 million in 1996 to 1998 to 102.5 million in 2009 to 2011, and the number of prescriptions for MSDs rose from approximately 996 million in 1996 to 1998 to about 2.1 billion in 2009 to 2011.^[2]^ MSDs are a leading cause of disease burden in the United States and are second only to cancer in Australasia.^[3,4]^ MSDs have become a major public health problem globally.^[5]^ Given the rising burden of MSDs, early accurate diagnosis is very important.

In patients with MSDs, an accurate differential diagnosis is key to selecting appropriate treatments. Current diagnostic imaging modalities involves radiography, computed tomography (CT) scans, ultrasound imaging, or magnetic resonance imaging (MRI). However, current approaches for diagnosing MSDs often involve ionizing radiation, thus resulting in harmness to the test subjects. Moreover, classical approaches do not apply to all subjects and are even limited by working hours.^[6]^ In recent years, increasing studies have shown that infrared thermography (IRT) is useful in the diagnosis of MSDs.^[7–9]^ Apart from the advantages of non-contact, low cost, portability, and absence of radiation, IRT allows real-time scanning and precise skin surface measurement to examine temperature variations indicating disease.

The diagnostic accuracy of IRT in MSDs was previously studied, producing heterogeneous findings.^[10]^ This might be attributed to inadequate studies for analysis, the methodological differences, different random errors, and different diagnostic boundaries. These differences may be caused by various factors, mainly caused by the insufficient design of diagnostic accuracy studies.^[11]^ Simultaneously, the features of the infrared camera and the time of use of the equipment could explain the low value of diagnostic accuracy.

Given that current evidence is inconclusive on IRT's diagnostic accuracy, we performed this study to determine the diagnostic accuracy of IRT for MSDs.

## 2 Methods

### 2.1 Design and registration

A meta-analysis of diagnostic test accuracy will be carried out as per the Preferred Reporting Items for Systematic Reviews and Meta-Analyses (PRISMA-P) and PRISMA of Diagnostic Test Accuracy (PRISMA-DTA) guidelines.^[12,13]^ The study protocol has been registered on the prospective register of systematic review (PROSPERO); the registration number was CRD42020184867. The research question was developed using the PICO research framework.

### 2.2 Participants/Interventions

Participants will be adults (age: ≥18 years) with a confirmed diagnosis of MSDs, comparing IRT versus other diagnostic strategies.

### 2.3 Comparator

This study will be non-comparative.

### 2.4 Sources of data

The databases searched from inception to November 1, 2020, included MEDLINE, EMBASE, EBSCO, Cochrane Library, SCOPUS, Web of Science, CNKI, SinoMed, Wangfang, and airiti library. There will be no language restrictions. Also, we will carefully check reference lists of articles to identify additional eligible articles.

The key text words used for the search included “thermography,” “musculoskeletal injury,” and “diagnosis.” To widen the usage of these keywords, we will include their synonyms and form a set of terms in the form of a free text and terminology from each database; through this, the set intersection will be determined and will be the final result of the entry searches. We will tailor the format and search term combination to fit every electronic database. Search strategies in the MEDLINE (via PubMed) database are provided in Table 1.

**Table 1:** Search strategy for the MEDLINE electronic database using PubMed.

Strategy number	Search items
1	Search ((((Temperature Mapping[Title/Abstract]) OR Mapping, Temperature[Title/Abstract]) OR Mappings, Temperature[Title/Abstract]) OR Temperature Mappings[Title/Abstract]) OR “Thermography”[Mesh]
2	Search (((((((((((((((((((((((((((((((((((Injury, Repetition Strain[Title/Abstract]) OR Injuries, Repetition Strain[Title/Abstract]) OR Repetition Strain Injuries[Title/Abstract]) OR Strain Injuries, Repetition[Title/Abstract]) OR Strain Injury, Repetition[Title/Abstract]) OR Repetition Strain Injury[Title/Abstract]) OR Overuse Injury[Title/Abstract]) OR Injuries, Overuse[Title/Abstract]) OR Injuries, Overuse[Title/Abstract]) OR Overuse Injuries[Title/Abstract]) OR Repetitive Motion Disorders[Title/Abstract]) OR Disorder, Repetitive Motion[Title/Abstract]) OR Disorders, Repetitive Motion[Title/Abstract]) OR Motion Disorder, Repetitive[Title/Abstract]) OR Motion Disorders, Repetitive[Title/Abstract]) OR Repetitive Motion Disorder[Title/Abstract]) OR Repetitive Strain Injury[Title/Abstract]) OR Injuries, Repetitive Strain[Title/Abstract]) OR Injury, Repetitive Strain[Title/Abstract]) OR Repetitive Strain Injuries[Title/Abstract]) OR Strain Injuries, Repetitive[Title/Abstract]) OR Strain Injury, Repetitive[Title/Abstract]) OR Overuse Syndrome[Title/Abstract]) OR Overuse Syndromes[Title/Abstract]) OR Repetitive Stress Injury[Title/Abstract]) OR Injuries, Repetitive Stress[Title/Abstract]) OR Injury, Repetitive Stress[Title/Abstract]) OR Repetitive Stress Injuries[Title/Abstract]) OR Stress Injuries, Repetitive[Title/Abstract]) OR Stress Injury, Repetitive[Title/Abstract]) OR Trauma Disorders, Cumulative[Title/Abstract]) OR Cumulative Trauma Disorder[Title/Abstract]) OR Disorder, Cumulative Trauma[Title/Abstract]) OR Disorders, Cumulative Trauma[Title/Abstract]) OR Trauma Disorder, Cumulative[Title/Abstract]) OR “Cumulative Trauma Disorders”[Mesh]
3	Search (((((((((((Diagnoses[Title/Abstract]) OR (Diagnoses[Title/Abstract] AND Examinations[Title/Abstract])) OR (Examinations[Title/Abstract] AND Diagnoses[Title/Abstract])) OR Postmortem Diagnosis[Title/Abstract]) OR Diagnoses, Postmortem[Title/Abstract]) OR Diagnosis, Postmortem[Title/Abstract]) OR Postmortem Diagnoses[Title/Abstract]) OR Antemortem Diagnosis[Title/Abstract]) OR Antemortem Diagnoses[Title/Abstract]) OR Diagnoses, Antemortem[Title/Abstract]) OR Diagnosis, Antemortem[Title/Abstract]) OR “Diagnosis”[Mesh]
4	#1 AND #2 AND #3

### 2.5 Study selection

**Figure 1:**
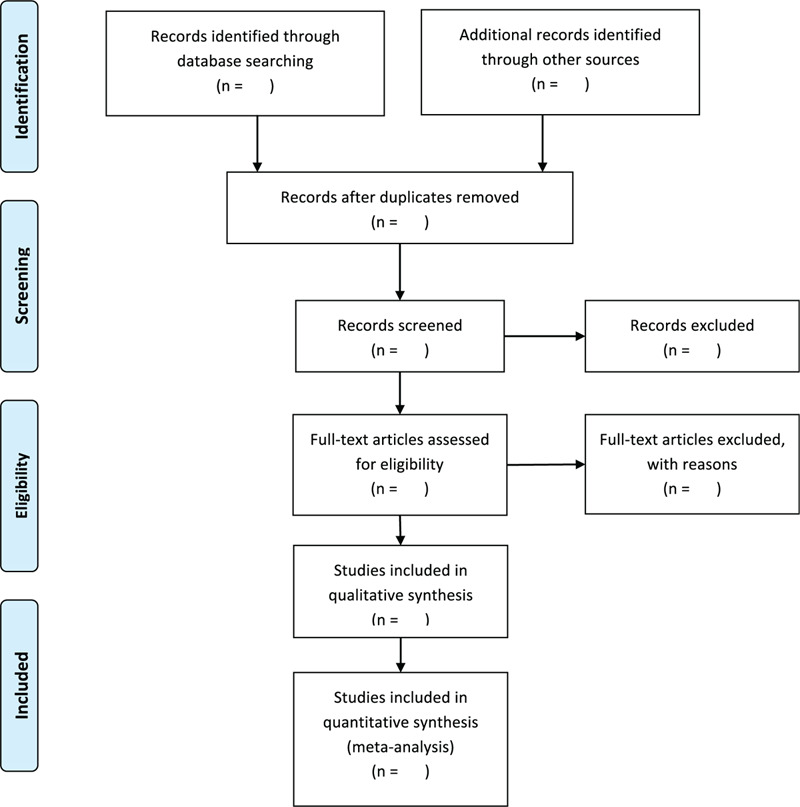
PRISMSA flow diagram of studies selection process. PRISMA = Preferred Reporting Items for Systematic Reviews and Meta-Analyses.

Two researchers (XYL and JLL) will conduct a literature search independently and select relevant articles in the databases mentioned above. Then, the results of literature searches will be transferred into EndNote, eliminating duplicated entries. Two researchers will peruse the articles independently with special attention to the titles, abstracts, and keywords and decide on their eligibility as per the agreed criteria. Contact will be made with the relevant studies’ authors to access additional information, particularly in cases of missing data. Any disagreement will be settled by a third reviewer (RS). Reasons for article exclusion will be recorded. The flowchart of the selection process is presented on Fig. 1.

The following inclusion criteria will be applied: study design: studies in medical settings describing diagnostic accuracy with adequate information to enable the calculation of 2 × 2 tables without setting development time/monitoring limitations; population: adult subjects (aged 18 years and older) diagnosed with MSDs; Index test: IRT (using any apparatus or means); Reference standard: the clinical interpretation of image diagnostic exploratory tests (e.g., radiography, MRI, CT, or ultrasound scan).

The following exclusion criteria will be applied: do not analyze IRT value on MSD diagnosis; not fit into the modality of scientific production (for example, review studies, letters to the editor; case reports and series; laboratory studies); not a human study; not provide data necessary for calculating a 2 × 2 table for specificity and sensitivity; lack of full text or inability to obtain it.

### 2.6 Quality and risk of bias assessment

The Quality Assessment of Diagnostic Accuracy Studies (QUADAS-2) tool will be utilized for methodological evaluation.^[14]^ Concerns regarding applicability and risk of bias will be examined synchronously in QUADAS-2. Two researchers (XYL and YJZ) will conduct an independent quality assessment using the tool; a third independent researcher will be available to settle disagreements.

### 2.7 Data extraction

Two researchers (XYL and JLL) will independently extract the data as follows: study information: authors, country, year of publication, and journal; study characteristics: research periods, fundings, research types, research methods; population characteristics: participants, ages, sexes, disqualified participants, study dropouts, and sampling techniques; a description of the diagnostic tests: index tests, reference, and comparator tests; timing and flow: time interval, interventions, and the training of researchers on the interpretation of the diagnostic tests; outcomes to estimate the test accuracy: a cross-tabulation of index test with a reference standard (2 × 2 table), sensitivity, positive likelihood ratio, specificity, negative likelihood ratio, diagnostic odds ratio (OR), and a receiver operating characteristic (ROC) curve. A third reviewer will settle discrepancies during data extraction.

### 2.8 Data synthesis and analysis

For the first step in data synthesis, the information will be collected from each selected study, and descriptive statistics calculated for each primary study. Kappa index (95% CI) was employed to calculate interrater reliability. Data analyses will be completed using STATA software (v14.0; StataCorp LP, College Station, TX). A probability (*P*) value of <.05 will define statistical significance. Egger's linear regression test and funnel plot will be employed to estimate publication bias.

#### 2.8.1 Diagnostic accuracy

We will extract the adequate data to build 2 × 2 tables for every study. Using these tables, we will calculate estimations of sensitivity (TP/[TP + FN]) and specificity (TN/[TN + FP]) (with 95% CI). Then the STATA 14.0 software (StataCorp LP) will be used to develop forest plot and summary receiver operating characteristic (SROC) curves. The random-effect model will be utilized to combine the specificity, sensitivity, positive and negative likelihood ratio (PLR and NLR), and diagnostic odds ratio (DOR) estimates due to heterogeneity, and analyze the SROC curves. The *y* axis represents sensitivity, whereas the *x* axis indicates specificity in the SROC curves. Each data point indicates one individual study. The area under the curve (AUC) will represent the final comparison. The AUC were categorized as follows: excellence, 0.90 to 1; good, 0.80 to 0.90; fair, 0.70 to 0.80; poor, 0.60 to 0.70 (poor); and failure, 0.50 to 0.60.

#### 2.8.2 Heterogeneity analysis

Cochran Q-statistic will be utilized to evaluate the heterogeneities and variations within and between the studies. Subsequently, *I*^2^ (range: 0% to 100%) was used to quantify the impact of heterogeneity among the trials in each analysis. *I*^2^ values categorized heterogeneity follows: 0% to 40%, not important; 30% to 60%, moderate; 50% to 90%, substantial; 75% to 100%, considerable. In the case that we find a significant amount of unexplained heterogeneity, we will explore possible causes through subgroup analysis and perform stratified meta-analyses to exclude these sources.

#### 2.8.3 Subgroup analysis

Subgroup analysis will be performed in cases where the studies included are not adequate. Additionally, we will conduct meta-regression. These analyses will consider factors such as age, country of publication, duration of index tests, and MSD risk factors and will be carried out to evaluate possible sources of heterogeneity in the present study. Covariates to be utilized include sample size, diagnostic criteria, and study design.

### 2.9 Patient and public participation

This study will not directly involve patients or the public.

## 3 Discussion

The recent impressive advancement of IRT in the quality of imaging and increasing computer processing power, the application of IRT in medicine is gaining popularity in disease diagnosis.^[15–19]^ In addition, the application of IRT to the diagnosis of MSDs can reduce the medical cost and radiation damage to humans. This may contribute to the development of health systems, especially in rural and remote areas, as well as bring new opportunities and challenges to the development of clinical guidelines.

Although studies are indicating that IRT enhances the precision of MSDs diagnosis, the current proof is inconclusive.^[20,21]^ Thus, suitable methods and tools for quality assessment will be employed to systematically examine the models used in MSD diagnosis to provide reliable proof for clinical practice. It is noteworthy that in-depth scientific investigation is required to establish a diagnostic approach that can precisely reveal the meaning of the evidence provided. Therefore, this study is to assess the diagnostic accuracy of IRT for MSDs. The results are expected to provide an interdisciplinary field of current research and new directions for diagnosing MSDs injuries.

The main limitation of the present study is that its findings may be affected by the small sample size regarding the number of studies included and thus may not have a universal application.

### 3.1 Patient and public involvement

Patients and public will not be involved in this study.

## Author contributions

**Data curation:** Xiaoyu Li, Jiali Lou.

**Formal analysis:** Xiaoyu Li, Yajun Zhang.

**Funding acquisition:** Jianqiao Fang.

**Methodology:** Yajun Zhang, Haiju Sun, Xiaofen He.

**Project administration:** Jianqiao Fang.

**Resources:** Haiju Sun.

**Software:** Xiaoyu Li, Yajun Zhang, Haiju Sun.

**Writing – original draft:** Xiaoyu Li.

**Writing – review & editing:** Yongliang Jiang, Jianqiao Fang.
